# The whole is greater than the sum of its parts: Long‐read sequencing for solving clinical problems in haematology

**DOI:** 10.1111/jcmm.17961

**Published:** 2024-01-23

**Authors:** Carlos Bravo‐Perez, Rosa Cifuentes‐Riquelme, Jose Padilla, Maria E. de la Morena‐Barrio, Francisco J. Ortuño, Pedro Garrido‐Rodríguez, Maria L. Amigo, Inmaculada Heras, Vicente Vicente, Maria L. Lozano, Raul Teruel‐Montoya, Belen de la Morena‐Barrio, Javier Corral

**Affiliations:** ^1^ Servicio de Hematología, Hospital Universitario Morales Meseguer, Centro Regional de Hemodonación Universidad de Murcia, IMIB‐Pascual Parrilla, CIBERER‐ISCIII Murcia Spain

**Keywords:** acute leukaemia, chromosomal rearrangements, long‐read sequencing, nanopore sequencing, molecular diagnostics, oncogenes

Structural variations (SVs) are common in haematological neoplasms.^1^ Although most SVs have canonical breakpoints, virtually all have atypical rearrangements that can be of difficult diagnosis. High‐throughput sequencing (HTS) has improved the study of atypical SVs, complementing cytogenetics. However, the short reads (<150 bp) generated by HTS might lead to inaccurate mapping.^2^ This may not suppose a mere technical pitfall, but a problem to be faced, to guarantee the monitoring and guided‐treatment of patients bearing atypical SVs when standard procedures are ‘blind’ to them.

Inversion of chromosome 16, inv(16)(p13q22)/t(16;16)(p13;q22), found in 5%–7% of de novo acute myeloid leukaemia (AML), joins *CBFB* (16q22) and *MYH11* (16p13) genes to form a chimeric oncoprotein that sequesters RUNX1. Up to 95% of cases with AML‐inv(16) carry three recurrent breakpoints involving *CBFB* exon5, and *MYH11* exon33, exon29 or exon28, resulting in type A, D or E transcripts, respectively.^3^ To the best of our knowledge, at least 10 additional *CBFB::MYH11* transcripts with non‐canonical breakpoints have been reported (Table 1).^4^, ^5^, ^6^, ^7^, ^8^, ^9^, ^10^, ^11^, ^12^, ^13^ However, direct characterization of inv(16) at the genomic level has rarely been performed. In this context, third‐generation HTS based on long reads, such as nanopore sequencing, could be excellent tools for the identification and comprehensive analysis of atypical SVs.^2^

**TABLE 1:** Summary of reported *CBFB::MYH11* transcripts with exonic/cDNA breakpoints and estimated protein fusion length.

Transcript type	Breakpoints (Exon)		Breakpoints (cDNA)		Inserted nucleotides at breakpoint	Fusion protein length (residues)	Reference
	CBFB^a^	MYH11^b^	CBFB^a^	MYH11^b^			
A	5	33	495	4579	No	625	[4]
B	5	32	495	4366	No	696	[5]
C	5	31	495	4186	No	756	[6]
D	5	29	495	3858	No	865	[4]
E	5	28	495	3652	No	934	[4]
F	4	33	399	4579	No	593	[7]
G	4	29	399	3858	No	833	[7]
H	4	28	399	3746	5 bp	856	[7]
I	4	34	399	4792	No	522	[8]
J	5	30	495	3977	No	830	[9]
K	6	28	534	3804	40 bp (exon 6)	549	[10]
Rowe et al.	5	32	495	4454	7 bp	655	[11]
Albano et al.	4	33	399	4708	21 bp (399 + 3979_4000ins)	543	[12]
Kurata et al.	5	26	495	3443	13 bp	994	[13]
This study	4	26	399 + 5751	3581	62 bp (399 + 5689_5751ins)	981	—

^a^ *CBFB* reference sequence: NM_022845.3.

^b^ *MYH11* reference sequence: NM_002474.3.

In this study, we characterize by nanopore sequencing a novel inv(16) in AML. It was detected in a 25‐year‐old woman admitted to our centre because mucosal bleeding. The hemogram revealed thrombocytopenia (18.0 × 10^9^/L) and monocytosis (5.4 × 10^9^/L), with 38% blasts in the blood smear. Bone marrow (BM) aspirate showed the presence of 58% blasts with morphologic and immunophenotypic features of myelomonocytic differentiation. In addition, 15% of atypical eosinophils and their precursors were identified (Figure 1A).

**FIGURE 1:**
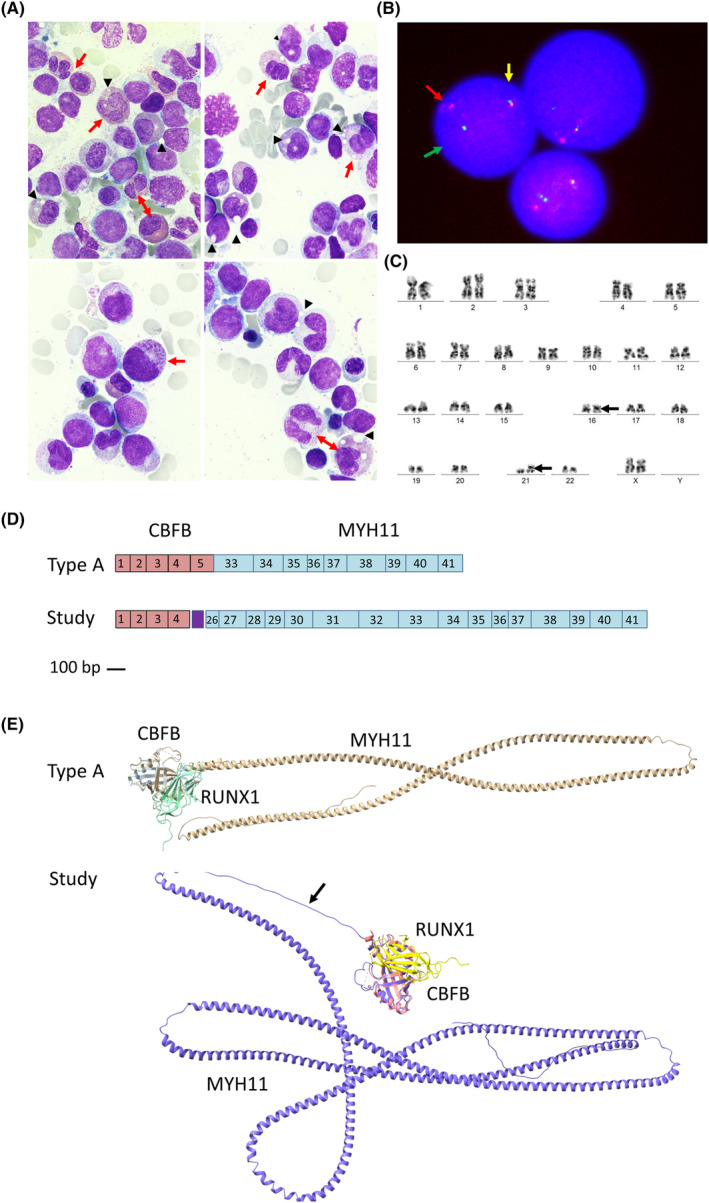
Characterization of atypical inv(16)(p13.1q22), CBFB::MYH11. (A) Bone marrow smear with May Grunwald‐Giemsa (×1000, lower panels are additionally magnified) showing infiltration by myelomonocytic blasts and 15% abnormal eosinophils and their precursors, most of them with abnormal proeosinophilic granules with a characteristic barely pinkish‐orange staining (red arrows). In addition, a remarkable cytoplasmic vacuolization was observed, which was not stained (black arrowheads). (B) FISH of bone marrow cells using the CBFB break apart probe (LSI CBFB Dual Color Break Apart Probe; MetaSystems, Germany) showing one fusion signal (unsplit CBFB, 16q21‐22, yellow arrow), one green signal (split CBFB, 5′ end, green arrow) and one red signal (split CBFB, 3′ end, red arrow). (C) Giemsa‐banded karyotype of bone marrow cells at diagnosis: 46,XX,inv(16)(p13q22),add(21)(p10)[14]/46,XX[6]. (D) Graphical representation of the common type A and the new atypical transcript found in this study type A. Scale representation (scale bar 100pb) of the contribution of the exons of CBFB (red boxes) and MYH11 (blue boxes) to each transcript. The linker peptide of the atypical transcript not belonging to exonic sequences is shown (purple boxes). (E) AlphaFold in silico modelling of the CBFB::MYH11 fusion proteins, and RUNX1 interaction, of the common type A and of the new aberrant inversion found in this study. The linker peptide is indicated with an arrow. Results were visualized with UCSF ChimeraX v1.4.

FISH showed inv(16)(p13q22). Karyotype confirmed inv(16) and add21(p10) (Figure 1B,C). However, RT‐PCR in BM RNA with commercial primers designed to detect the canonical *CBFB::MYH11* transcripts (QuantiTec probe RT‐PCR kit, Qiagen, Germany, Table S1) gave negative results. A myeloid HTS panel (Oncomine Myeloid Research Assay‐Chef Ready, Thermofisher, MA, USA) using Ion S5 identified a *CBFB::MYH11* rearrangement in 192/24685 RNA reads involving *CBFB* exon4 and *MYH11* exon28. In addition, two pathogenic variants were identified in *ZRSR2* (c.1147C > G p.Pro383Ala) and *KRAS* (c.38G > A p.Gly13Asp).

The patient was diagnosed with AML‐inv(16). BM re‐evaluation after 3 + 7 induction showed persistence of 29% blasts. Salvage chemotherapy with FLAG‐IDA (PETHEMA‐CBF‐2016) was then administered. Subsequently, haematologic and cytogenetic complete remission (CR) was achieved. However, flow cytometry revealed positive minimal residual disease (MRD 0.20%). The patient then underwent allogeneic haploidentical haematopoietic stem cell transplantation (HSCT) from a sibling. Since day +28 post‐transplant and to date (4 years later), she is in CR with negative MRD by flow cytometry and has complete donor chimerism.

The patient fortunately achieved a durable remission. However, at the diagnostic level, this scenario revealed a problem to be solved: the impossibility of monitoring the atypical CBFB::MYH11 transcript using standard diagnostic systems, including RT‐PCR kits. The root of this difficulty was the incomplete molecular characterization of the inv(16). Cytogenetics and Oncomine RNA sequencing identified an inv(16) and a CBFB::MYH11 transcript, but did not accurately characterized them.

Therefore, we performed genomic long‐read sequencing using nanopore technology. The study was performed on a MinION instrument (Oxford Nanopore Technologies [ONT], UK) using adaptive sampling, a computationally driven method for targeted sequencing of individual DNA molecules based on the identity of an initial set of approximately 450 bp, which are real‐time compared to a reference sequence to decide whether a given molecule should be sequenced further or rejected. The genome coordinates for enrichment were chr16:66297445–67,900,545 and chr16:14968145–16,559,989, which contain the *CBFB* and *MYH11* genes, respectively. The library was prepared using the kit SQK‐LSK109 (ONT, UK). The run (20 h) yielded 10Gb. Mean coverage in the region of interest was 3.6X, while it was 1.3X in the rest of the genome. Basecalling was done with Guppy 6.4.2 using FAST. An in‐house multimodal pipeline including Sniffles software,^2^ found four reads supporting a 51 Mb inversion (Figure S1). De novo assembly of these reads described an atypical breakpoint affecting c.399 + 5751 of *CBFB* intron4 and c.3581 *MYH11* exon26, results discordant with the data initially provided by Oncomine. Genomic PCR covering the inversion (Table S2) and Sanger sequencing confirmed the breakpoint observed by nanopore: seq[GRCh38] inv(16)(p13q22) chr16:g.[15,735,438–67,072,549 inv] (Figure S1).

HSF v3.1 and NetGene2 v.2.42 were used for prediction of splicing. A very strong acceptor signal was identified in *CBFB* intron4, surrounded by silencer splicing signals (Figure S2). However, in the fusion gene, the *MYH11* exon26, located only 62 bp from this intronic acceptor site, provided counteracting enhancer splicing signals (Figure S2). Furthermore, the use of this cryptic intronic acceptor site would preserve the *MYH11* open reading frame. To test our prediction, RT‐PCR was performed on BM RNA at diagnosis using the primers and conditions listed in Table S2. This assay yielded only the expected 235 bp amplicon in the patient, and Sanger sequencing confirmed the predicted fusion. A nested RT‐PCR was also designed to increase sensitivity and evaluate MRD in patient's serial samples during treatment. This analysis did not detect the fusion transcript after HSCT, confirming the favourable evolution of the case (Figure S2).

The resulting chimeric protein might contain 981 residues: 133 from *CBFB* (exon 1–4), 20 from the retained region of *CBFB* intron 4, and 829 from *MYH11* (exon 26–41). Notably, it represents one of the longest *CBFB::MYH11* described to date, and the one with the highest number of residues at the fusion point not corresponding to *CBFB* or *MYH11* exons (Table 1, Figure 1D).

Finally, we used Alphafold for in silico modelling of the atypical oncoprotein,^14^ and compared it with the common type A form. As shown in Figure 1E, in both fusion proteins, the N‐terminus encoded by *CBFB* was able to bind RUNX1, while the C‐terminus encoded by *MYH11* formed a long α‐helix. However, the length of the α‐helix was extremely different: the one resulting from the new transcript was almost twice as long as the canonical oncoprotein. In addition, *CBFB* intron4 encoded a long, unstructured loop connecting the CBFB globular domain to the MYH11 helix.

Using nanopore sequencing and a novel methodology, adaptive sampling, which does not require expensive equipment, in a fast and economical process, we characterized a new atypical inv(16) with nucleotide resolution in a patient with de novo AML. It remains to be determined whether this oversized oncoprotein might influence AML‐inv(16) pathogenesis. Unlike most cases with non‐A type transcripts that have been reported, and despite having one of the most genuine *CBFB::MYH11* fusions, the patient showed many of the features that can be expected of a bona fide AML‐inv(16). Interestingly, transcripts with an alternative 5′ breakpoint at *CBFB* exon4 (i.e. lacking *CBFB* exon5) have been associated with AML‐inv(16) without myelo‐monocytic differentiation.^3^, ^4^, ^5^ Shigesada et al. suggested that longer transcripts at the expense of the MYH11 N‐terminus might have uncoiled, flexible regions N‐terminal to the coiled‐coil polymerization domains, which might help CBFB bind RUNX1 with higher affinity.^3^ In this sense, we could speculate that, in contrast to other transcripts lacking *CBFB* exon5, the oversized N‐terminal region of MYH11 in our case, separated from CBFB by an unfolded linker of 20 residues, could lead to the type A‐like leukaemic phenotype by (1) cooperation with the truncated CBFB to overcome the exon5 deletion and bind RUNX1 and (2) straight fibre formation by an extra‐long coiled‐coil domain.

It is even more difficult to speculate on the clinical implications of this new aberrant inversion in an isolated patient. Case reports and small series suggest that non‐A type transcripts are associated with worse clinical outcomes than A type transcripts. However, this assumption has been challenged by others. In addition, the level of complexity may be increased by concomitant chromosomal or gene abnormalities.^3^, ^4^

Finally, our findings may have therapeutic implications. Genomic mapping of the inversion allowed the discovery of the mechanism leading to the fusion protein: the use of an intronic cryptic acceptor splice site. Our findings provide the rationale to test novel therapeutic approaches targeting splicing in preclinical models of AML.

In conclusion, this study reveals new evidence for the diagnostic potential of long‐read sequencing to offer a ‘whole picture’ of SVs that may not be adequately represented by the ‘sum of parts’ of short‐read HTS methods.^15^ Moreover, it illustrates how the complete characterization of the driver defect of any haematological neoplasm could not only allow us to refine disease monitoring and patient management, thereby solving clinical problems, but also provide deeper insights into the underlying pathogenic mechanisms. Ultimately, new and specific therapies may be identified based on these findings.

## AUTHOR CONTRIBUTIONS

**Carlos Bravo‐Perez:** Conceptualization (equal); data curation (equal); formal analysis (lead); investigation (equal); methodology (equal); visualization (equal); writing – original draft (lead); writing – review and editing (lead). **Rosa Cifuentes‐Riquelme:** Conceptualization (equal); data curation (equal); formal analysis (equal); investigation (equal); methodology (lead); visualization (equal); writing – original draft (equal). **José Padilla:** Data curation (equal); investigation (equal); methodology (equal); software (equal); writing – original draft (supporting); writing – review and editing (supporting). **Maria Eugenia de la Morena‐Barrio:** Conceptualization (equal); investigation (equal); methodology (equal); software (equal); validation (equal); visualization (equal); writing – original draft (supporting); writing – review and editing (supporting). **Francisco Ortuño:** Investigation (equal); methodology (equal); resources (equal); visualization (equal); writing – original draft (supporting); writing – review and editing (supporting). **Pedro Garrido‐Rodríguez:** Data curation (equal); investigation (equal); methodology (equal); software (equal); visualization (equal); writing – original draft (supporting); writing – review and editing (supporting). **MariLuz Amigo:** Data curation (equal); resources (equal); writing – original draft (supporting); writing – review and editing (supporting). **Inmaculada Heras:** Data curation (equal); resources (equal); writing – original draft (supporting); writing – review and editing (supporting). **Vicente Vicente:** Conceptualization (equal); supervision (equal); writing – original draft (supporting); writing – review and editing (supporting). **María Luisa Lozano:** Conceptualization (equal); supervision (equal); writing – original draft (supporting); writing – review and editing (supporting). **Raul Teruel‐Montoya:** Data curation (equal); investigation (equal); methodology (equal); resources (equal); writing – original draft (supporting); writing – review and editing (supporting). **Belen de la Morena‐Barrio:** Data curation (equal); methodology (equal); resources (equal); writing – original draft (supporting); writing – review and editing (supporting). **Javier Corral:** Conceptualization (lead); funding acquisition (lead); project administration (lead); supervision (lead); writing – original draft (lead); writing – review and editing (lead).

## FUNDING INFORMATION

The study was funded by Instituto de Salud Carlos III (ISCIII) through the project ‘PMP21/00052’, which was co‐funded by the European Union‐Next Generation EU. Cifuentes‐Riquelme R has a PFIS contract. de la Morena‐Barrio ME has a Ramon y Cajal (RYC2021‐031000‐I) from Ministerio de Ciencia e Innovación. Bravo‐Pérez C has a Juan Rodés fellowship (JR22/00041). de la Morena‐Barrio B has a postdoctoral contract from CIBERER. Garrido‐Rodríguez P has a predoctoral contract from CIBERER.

## CONFLICT OF INTEREST STATEMENT

The authors declare no conflicts of interest.

## Supporting information


Data S1.

## Data Availability

For original data, please contact javier.corral@carm.es.
