# Supplementary material for: The whole is greater than the sum of its parts: Long‐read sequencing for solving clinical problems in haematology
Source: J Cell Mol Med. 2024 Jan 23;28(3):e17961. doi: 10.1111/jcmm.17961 (PMC10844759; doi:10.1111/jcmm.17961)
Supplement: Supplementary file 1 — Data S1. [file JCMM-28-e17961-s001.docx]

**SUPPLEMENTARY MATERIAL**

**Table of contents**

**Supplementary Figures**

**Figure S1…………………………………………………………………………………………………………………..…………………2**

**Figure S2……………………..………………………………………………………………………………………………………………4**

**Supplementary Tables**

**Table S1……………………………………………………………………………………………….…………………………….………6**

**Table S2……………………………………………………………………………………………….…………………………….………7**

**
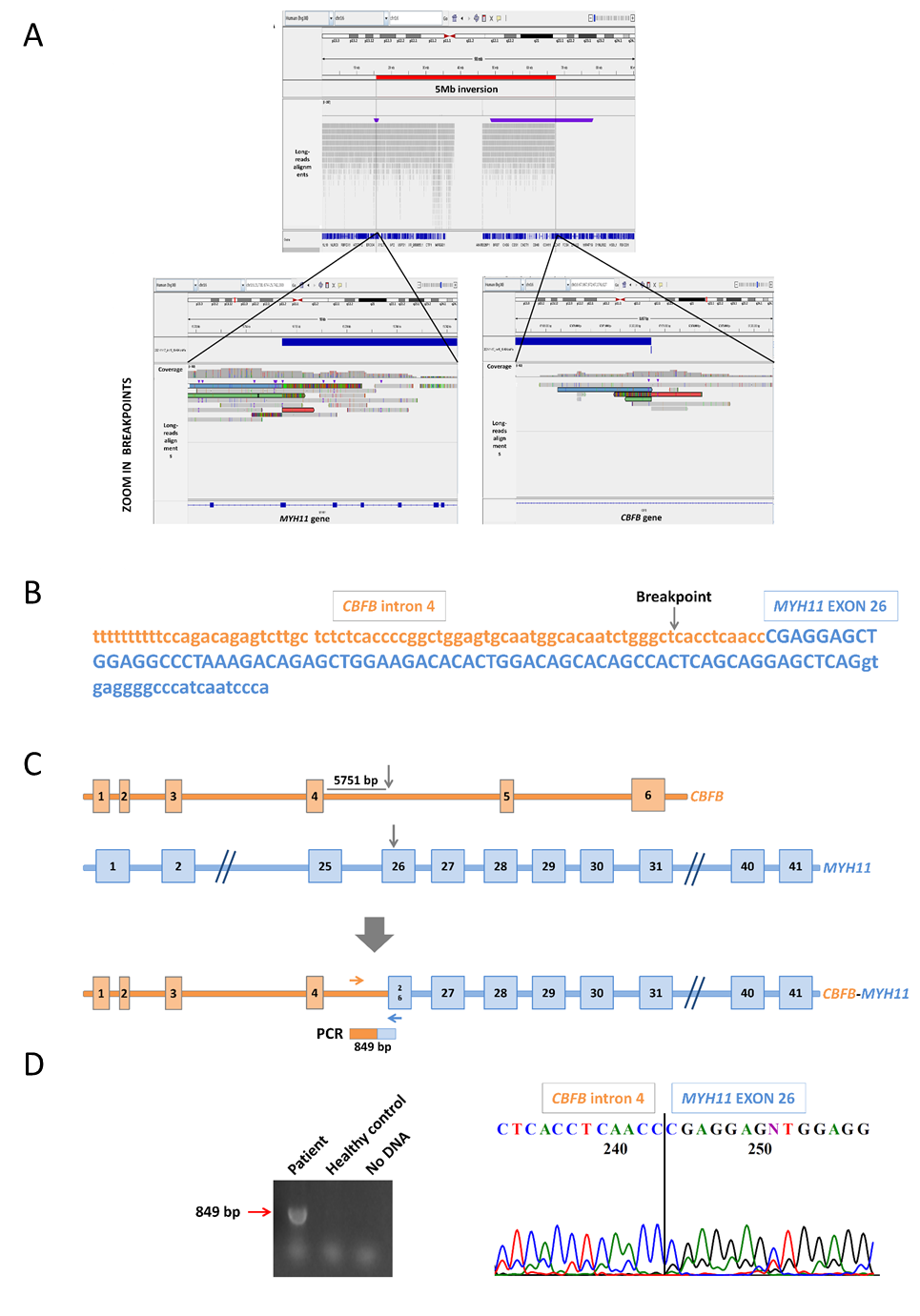
**

***Figure S1. Nanopore sequencing and detection of an atypical 51Mb inversion in chromosome 16 involving CBFB and MYH11.*** *A) The whole inversion is highlighted in red. Zoom in on specific breakpoints of the inversion in MYH11 and CBFB genes. B) Sequence of the breakpoint obtained by de novo assembly. C) Schematic representation of the genomic breakpoint in CBFB and MYH11 genes and the resulting fused CBFB::MYH11 gene. D) Validation by specific PCR using primers designed by the de novo assembly sequence of the inversion at genomic level. The electropherogram of the sequence covering the breakpoint is also shown.*


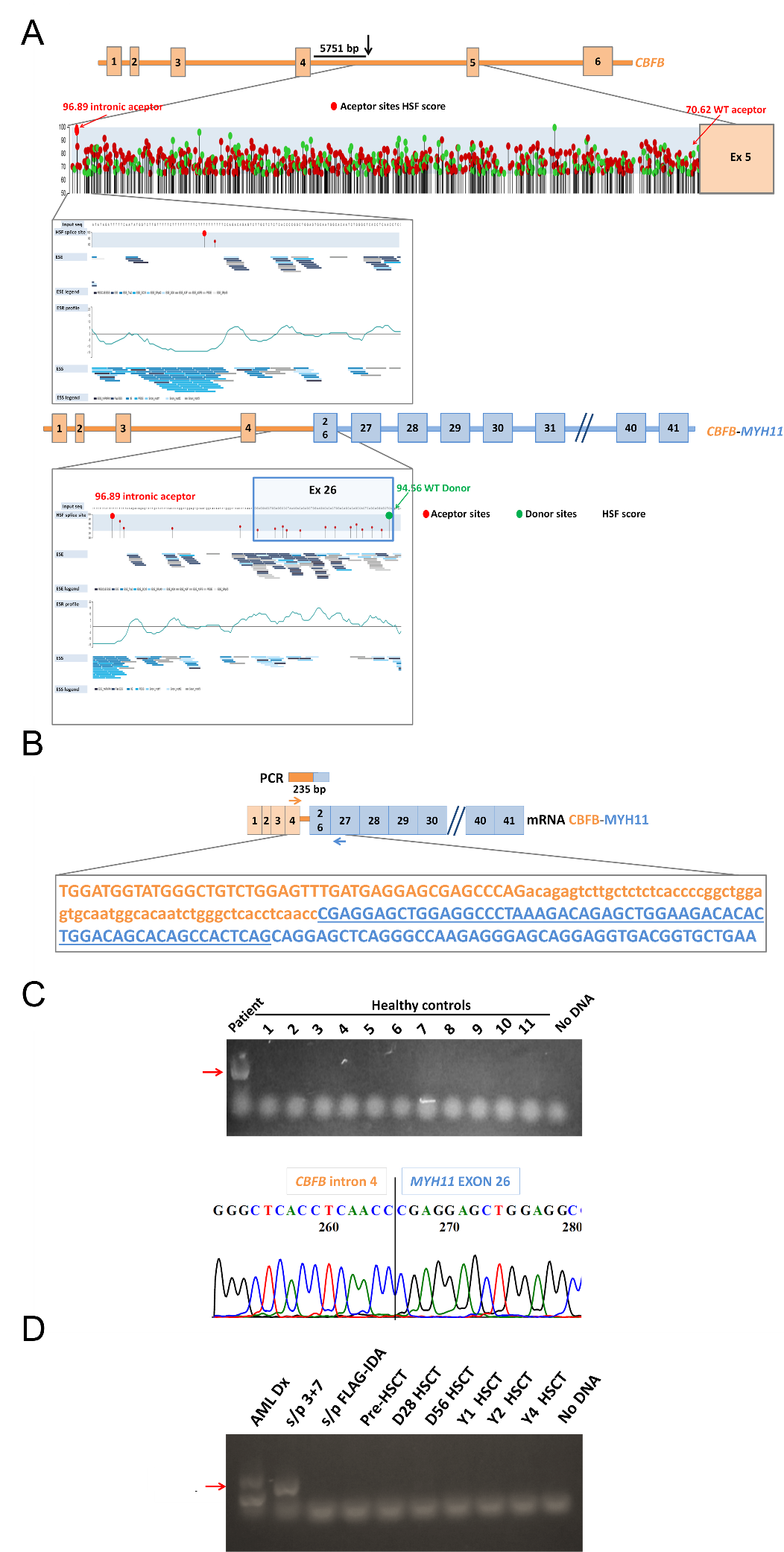


***Figure S2. Characterization of the splicing mechanism underlying the atypical CBFB::MYH11 transcript.*** *A) Human Splicing Finder predictions of splicing signals in the wild type and fusion genes. The score of the strongest and physiological donor and acceptor sites are indicated. Potential exonic splicing enhancers (ESE), exonic splicing silencers (ESS) and exonic splicing regulators (ESR) are also shown. B) Predicted structure of the fusion transcript. C) Validation by specific PCR using primers designed by the de novo assembly sequence of the inversion at the genomic level. The electropherogram of the sequence covering the breakpoint is also shown. D) Validation of the fusion transcript by RT-PCR and sequencing in the patient at diagnosis. Follow-up of the fusion transcript by nested RT-PCR in the patient during 4 years.*

***Table S1. Primers used to identify by RT-PCR the common types A, D and E CBFB-MYH11 fusion transcripts.***

| **GENE** | **NAME** | **SEQUENCE (5’ - 3’)**  **PRIMERS AND PROBES** | **CONDITIONS** |
| --- | --- | --- | --- |
| FUSION GENE *CBFB-MYH11* | INV(16)-F | CATTAGCACAACAGGCCTTTGA | cDNA Synthesis: 50ºC→30' (2´5 ºC/s)  Enzyme Activation: 95ºC→15' (4´8 ºC/s)  Amplification 45 cycles:  95ºC → 15" (4´8 ºC/s)  60ºC → 60" (2´5 ºC/s single acquisition)  Cooling: 4ºC -- 10" (2 ºC/s) |
|  | INV(16)A-R | AGGGCCCGCTTGGACTT |  |
|  | INV(16)D-R | CCTCGTTAAGCATCCCTGTGA |  |
|  | INV(16)E-R | CTCTTTCTCCAGCGTCTGCTTAT |  |
| *ABL* | ABL ENF1003 | TGGAGATAACACTCTAAGCATAACTAAAGGT |  |
|  | ABL ENR1063 | GATGTAGTTGCTTGGGACCCA |  |
| FUSION GENE *CBFB-MYH11* | INV(16)E-P | **6FAM**TCGCGTGTCCTTCTCCGAGCCT**BHQ1** |  |
| *ABL* | ABL ENPr1043 | **6FAM**CCATTTTTGGTTTGGGCTTCACACCATT**BHQ1** |  |

***Table S2. Specific primers designed to validate at genomic or RNA levels the*** ***CBFB-MYH11, Inv(16) (p13q22), characterized by nanopore sequencing in a patient with AML.***

| **Amplification** | **Name** | **SEQUENCE (5’ - 3’)** | **Length** | **Annealing T (ºC)** |
| --- | --- | --- | --- | --- |
| **Genomic** | CBFB-F2 | 5´CACTTCCATGGCCCAACACA 3´ | 849 bp | 60º |
|  | MYH11-R2 | 5´ GTTTGTCCCCTCTTCTGCCC3´ |  |  |
| **RNA** | CBFB_ex3F | 5´ GGCCACAGGAACCAATCTGTC 3´ | 235 bp | 60º |
|  | MYH11_ex27R | 5´ TGCTCTGTGAGCTCCTCCA 3´ |  |  |
